# Transgenic HepaRG cells expressing CYP2D6 as an improved model of primary human hepatocytes

**DOI:** 10.1002/prp2.939

**Published:** 2022-02-17

**Authors:** Shota Okuyama, Akari Mine, Teppei Nakamura, Yusuke Ohasi, Mayuko Seto, Masako Tada

**Affiliations:** ^1^ Stem Cells & Reprogramming Laboratory Department of Biology Faculty of Science Toho University Funabashi Chiba Japan

**Keywords:** CYP2D6, CYP3A4, HepaRG, live‐cell imaging, polymorphism, transgenics

## Abstract

CYP2D6 and CYP3A4, which are members of the cytochrome P450 superfamily of metabolic enzymes, play major roles in the metabolism of commonly available drugs. CYP3A4 is involved in the metabolism of 50% of drugs on the market, whereas CYP2D6 is involved in the metabolism of 25% of them. CYP2D6 exhibits a high degree of polymorphic nature in the human population, causing individual differences in CYP2D6 expression and enzymatic activity. Therefore, accurate prediction of drug metabolism and toxicity require a human adult hepatocyte cell model that mimics individual responses in the average population. HepaRG cells, a human hepatocellular carcinoma cell line, is the only cell line that can differentiate into hepatocyte‐like cells with high expression of CYP3A4 but poor expression of CYP2D6. To solve this problem, we developed transgenic HepaRG cell clones expressing either full‐length or spliced CYP2D6 at various levels with an easy monitoring system for *CYP2D6* expression in living cells under a fluorescent microscope. As *CYP2D6* mRNA, protein, and fluorescence intensity were closely correlated among transgenic HepaRG clones, fluorescence levels will provide a simple tool for quality assurance of CYP2D6‐expressing HepaRG cells. Thus, the package of transgenic HepaRG cell clones expressing CYP2D6 at various levels will provide an improved hepatocyte model that reflects the average or individual reactions in the human population for in vitro studies of drug metabolism and toxicity involving CYP2D6 and CYP3A4.

## Graphical abstract

HepaRG cells are the only cell line that can differentiate into hepatocyte‐like cells that highly express CYP3A4. We have constructed transgenic HepaRG cells that express CYP2D6 at various expression levels for the two splicing forms. These cells are equipped with systems that allow easy live cell monitoring of CYP2D6 RNA or protein expression level under a fluorescence microscope. These cell lines may be useful as model cells for human hepatocytes coexpressing CYP3A4 and CYP2D6.

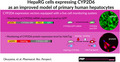

**Abbreviations**
CYP2D6: cytochrome P450 family 2 subfamily D member 6 enzymes
HLCs: hepatocyte‐like cells

**Significance Statement**

We describe the successful construction of transgenic HepaRG cells expressing CYP2D6 at various expression levels. These can be used as hepatocyte model cells that co‐express CYP3A4 and CYP2D6, reflecting individual differences in drug metabolism and sensitivity to toxicity in natural human hepatocytes.

## 1 INTRODUCTION

Many drugs are metabolized in the liver and undergo structural changes. CYP3A4 and CYP2D6 are major P450 enzymes, which participate in the metabolism of 50% and 25% of marketed drugs, respectively. *CYP3A4* transcription is induced by some drugs, causing hepatotoxicity and attenuating the pharmacological effects of those drugs and concomitant drugs, this drug–drug interaction is a serious hurdle in drug development. In contrast, *CYP2D6* is rarely transcriptional inducible, but it is polymorphic (showing mutations, copy number variation, and alternative splicing), resulting in individual differences in response to drugs.^1^ Therefore, there is a need for model cells that mimic primary human adult hepatocytes, reflecting real human metabolism and the polymorphic nature in the human population.

HepaRG cells are widely used as model cells for adult human hepatocytes because HepaRG cells are the only cultured cells that can be differentiated into adult human hepatocyte‐like cells (HLCs) highly expressing CYP3A4 to the levels of normal adult human hepatocytes.^2^ However, *CYP2D6* mRNA expression is limited in HepaRG cells to 1/35 to 50 of that in adult human hepatocytes, which constrains the use of HepaRG as model hepatocyte cells.^3^ Therefore, in this study, we developed transgenic HepaRG cells to act as HLCs that co‐express CYP3A4 and CYP2D6. We also tried to introduce the polymorphic nature of CYP2D6 in the transgenic HepaRG cells.

CYP2D6 metabolizes analgesics, antidepressants, antihypertensives, and anticancer drugs. In particular, the anticancer agent tamoxifen has therapeutic effects as a prodrug, and the metabolites of CYP2D6, 4‐OH‐tamoxifen and endoxifen, are highly effective in breast cancer treatment.^4^ Differences in the metabolic activity of CYP2D6 can seriously affect therapeutic efficiency and prognosis.^5^ CYP2D6 shows differences in protein structure due to selective splicing of exon 3 or exon 6.^6^ However, it remains unclear whether these splicing variants affect the metabolic function of CYP2D6.

In this study, therefore, we generated not only HepaRG cell clones expressing various levels of CY2D6, but also HepaRG cells expressing the full‐length or splicing variant of CYP2D6 to provide a package of HLCs that reflect both individual and population differences. In these living cells, the expression level of CYP2D6 can be easily monitored under conventional fluorescence microscopy.

## 2 MATERIALS AND METHODS

### 2.1 RNA extraction from cryopreserved primary human hepatocytes

Total RNA was extracted from human cryopreserved hepatocytes (AL174, male HEP187174; AL176, female HEP187176; AL224, female HEP187224) kindly provided by Biopredic International using the RNeasy Mini Kit (QIAGEN). Then, cDNA was synthesized using the SuperScript III First‐Strand Synthesis System for RT‐PCR (Life Technologies) and oligo‐dT primers.

### 2.2 TA cloning and DNA sequencing of the open reading frame of *CYP2D6*

The open reading frame (ORF) region of *CYP2D6* was amplified from hepatocyte cDNAs by ExTaq (Takara Bio) PCR using the primers listed in the Supporting Information (SI) Table 1. The PCR products were purified using the QIAquick Gel Extraction Kit (QIAGEN) and cloned into the pGEM‐T Easy vector (Promega). Nucleotide sequence analysis of 1,494 bp of DNA from AL174 and 1,341 bp of DNA from AL224 by Eurofins identified them as the ORFs of *CYP2D6* in transcript variant 1 (NM_000106.5) and transcript variant 2 (NM_001025161.2).

### 2.3 CYP2D6‐iGFP expression vector

To create pCMV‐*CYP2D6*‐*IRES*‐*GFP* (CYP2D6‐iGFP) expression vector, a DNA fragment of the *CYP2D6* ORF region was ligated between the CMV promoter and the *IRES*‐*GFP* using Ligation mix (TaKaRa) at 4°C overnight.

### 2.4 HaloTag‐CYP2D6 expression vector

HaloTag‐tagged CYP2D6 expression vector was purchased from Promega. The HaloTag ORF Clone FHC02139 ubiquitously expresses HaloTag‐*CYP2D6* under the CMV promoter. By adding a fluorescent ligand that specifically and covalently binds HaloTag to the culture medium,^7^ CYP2D6 protein in living cells was visualized by adding TMR ligands (Promega) into cell culture medium for 15 min followed by extensive washing.

### 2.5 Cell culture

Wild‐type (WT) HepaRG cells were purchased from Biopredic International. The cell seeding density and differentiation conditions have been described in detail previously^8^ and SI METHODS.

### 2.6 Generation of transgenic HepaRG cells

After linearizing the expression vectors with restriction enzymes, CYP2D6‐iGFP and Pgk‐neo vectors or HaloTag‐CYP2D6 and Pgk‐hyg vectors were co‐transfected into WT HepaRG cells in a 2:1 ratio using Lipofectamine LTX with Plus Reagent (Thermo Fisher Scientific) because none of the CYP2D6 expression vectors contain drug‐selective genes. Transgenic HepaRG cells expressing CYP2D6‐iGFP and HaloTag‐CYP2D6 were selectively cultured for 5 days in 710 growth medium supplemented with 800 µg/ml G418 (Sigma‐Aldrich) and 40 µg/ml hygromycin (Thermo Fisher Scientific), respectively. CYP2D6‐iGFP and HaloTag‐CYP2D6 transgenic HepaRG cell clones were positive for GFP and HaloTag TMR Ligand (Promega) staining, respectively. Cell colonies were cultured individually in 96‐well plates and then gradually expanded. After three to four passages in 24‐well and 6‐well plates, the first cell population cultured in 25 cm^2^ culture bottles was designated as passage 1, and cells from passage 2–3 were stored as primary cell stocks. HaloTag proteins were visualized by incubating cells for 15 min in a medium containing HaloTag TMR Ligand diluted at a ratio of 1:2000 and washed twice with PBS.^7^ F‐actin was stained with 1 × Phalloidin‐iFluor 488 Reagent (Abcam).

### 2.7 Fluorescence image analysis and PCR analysis

Fluorescence microscopic images were captured using a BZ‐9000 fluorescence microscope (Keyence) and an A1 confocal microscope (Nikon). Reagents, software, and methods used in this study have been described in detail previously^9^ in SI METHODS. Primer sets used for Genomic PCR and RT‐qPCR are listed in SI Table 1.

## 3 RESULTS

**FIGURE 1:**
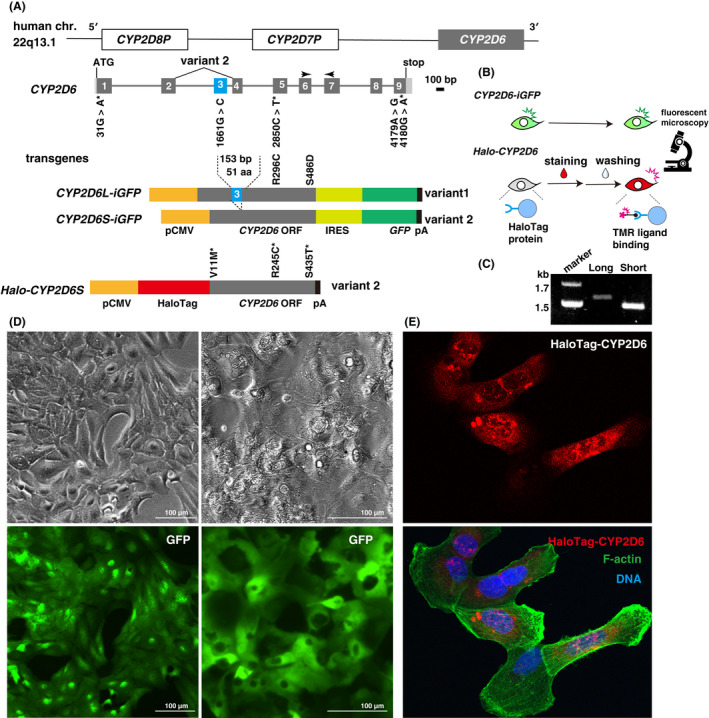
Transgenic HepaRG cells expressing CYP2D6. (A) Comparative display of the CYP2D6 genome, and CYP2D6‐iGFP and HaloTag‐CYP2D6 vectors. Mutations from sequence number NM_000106.5 are shown. Asterisk, mutations causing amino acid substitutions. L, long variant 1; S, short variant 2 skipping exon 3. (B) Schematic representation of the experimental procedure. (C) Plasmid DNAs of CYP2D6‐iGFP digested with EcoRI, showing the difference in the open reading frame length. (D) GFP fluorescence images show stable expression of CYP2D6 in growing (right) and differentiated (left) HepaRG cells. (E) Intracellular localization of TMR‐bound HaloTag‐CYP2D6 protein (red) and cytoplasmic F‐actin (green)

We isolated the ORF from primary adult hepatocytes. The ORF encodes the full‐length CYP2D6 transcript variant 1 (CYP2D6L) or the short CYP2D6 transcript variant 2 (CYP2D6S) lacking exon 3 (Figure 1A). The CYP2D6‐iGFP expression vector allows ubiquitous expression of *CYP2D6*‐*IRES*‐*GFP* mRNA under the CMV promoter and encodes two independent proteins, CYP2D6 and GFP, in relatively comparable amounts. Therefore, we planned to use it as a live‐cell monitoring system to predict the amount of CYP2D6 by green fluorescence intensity. In contrast, we planned to use the HaloTag‐CYP2D6 expression vector for quality assurance of the amount of CYP2D6 protein in the cell population just prior to its use in metabolic studies (Figure 1B). We isolated many transgenic HepaRG clones for each expression vector with different expression levels of *CYP2D6* variants (Figure 1C), as shown in Table 1. These transgenic clones generated HLCs similar to the original WT HepaRG cells and continuously expressed green fluorescence and HaloTag‐CYP2D6 before and after cell differentiation (Figure 1D). HaloTag‐CYP2D6 accumulated in endoplasmic reticulum (ER)‐rich perinuclear regions (Figure 1E), as CYP2D6 protein is an integral membrane protein and localizes to the ER.^10^

**TABLE 1:** List of transgenic HepaRG cells expressing human *CYP2D6* and PCR primers used in this study

Clone names	Open reading frame	No. of clones established	Nucleotide changes	Amino acid substitutions and alleles	
CYP2D6L‐iGFP	Full length	22	31G>A	V11 M	CYP2D6*35
			1661G>C		
			2580C>T	R296C	
			4179A>G		
			4180G>A	S486D	
CYP2D6S‐iGFP	Exon3 skipping	6	31G>A	V11 M	CYP2D6*35
			2427C>T	R245C	
			4026A>G		
			4027G>A	S435T	
HaloTag‐CYP2D6S	Exon3 skipping	4	31G>A	V11 M	CYP2D6*35
			2427C>T	R245C	
			4026A>G		
			4027G>A	S435T	

**FIGURE 2:**
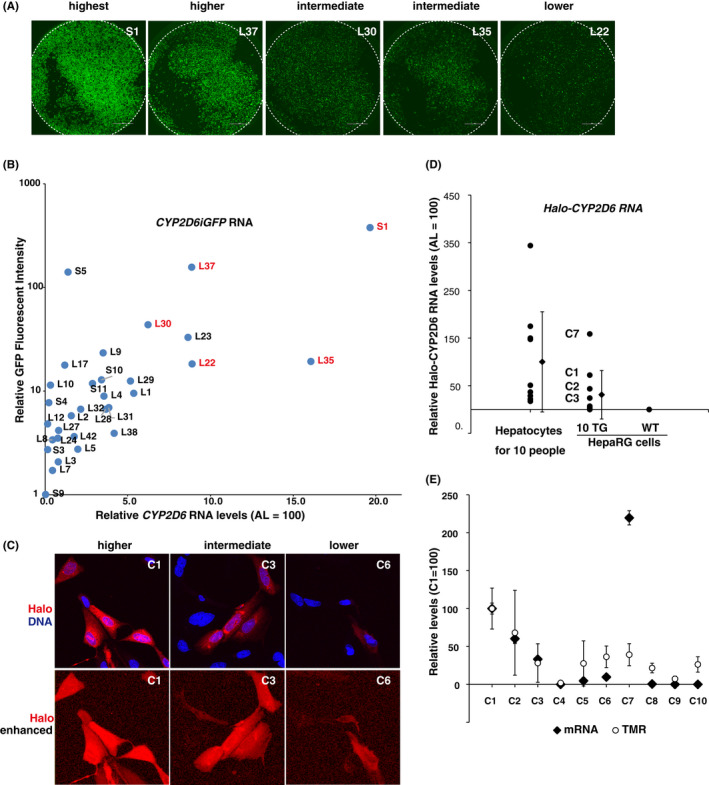
Various transgenic HepaRG cell clones to represent the polymorphism of *CYP2D6*. (A) Confocal microscopy images of representative HepaRG clones expressing CYP2D6‐iGFP. (B) Close correlation between GFP fluorescence levels and *CYP2D6* mRNA levels measured by RT‐qPCR in CYP2D6‐iGFP‐expressing HepaRG cells (n = 3). AL, adult human liver. (C) Confocal microscopy images of representative HepaRG clones expressing HaloTag‐CYP2D6 protein. (D) Comparison of *CYP2D6* mRNA expression levels measured by RT‐qPCR in 10 human primary livers and HaloTag‐CYP2D6 HepaRG clones. (E) In nine HaloTag‐CYP2D6 HepaRG clones, except for the clone C7, there is a correlation between red fluorescence levels corresponding to the amount of TMR ligands covalently bound to the HaloTag of CYP2D6 protein and *CYP2D6* mRNA levels measured by RT‐qPCR (n = 3). All transgenic clones analyzed here are listed in Table 1

HepaRG clones of CYP2D6iGFP showed various levels of green fluorescence, of which three clones, classified as higher, intermediate, and lower according to their expression levels, were shown in Figure 2A. The differences between clones may represent individual differences in the human population. The differences in fluorescence intensity between clones generally corresponded to differences in the expression levels of *CYP2D6* mRNA as measured by RT‐qPCR using day 7 cells after subculturing (Figure 2B). The highest expression level of *CYP2D6* mRNA was observed in the clone #1 of CYP2D6S‐IRES‐GFP HepaRG cells, with approximately 20% of adult human liver, whereas little *CYP2D6* mRNA expression was detected in WT HepaRG cells. Next, fluorescent cell images of HaloTag‐CYP2D6 HepaRG clones stained with red fluorescent‐labelled HaloTag ligand (TMR) are shown in Figure 2A. The red fluorescent intensity represents the amount of TMR ligand covalently bound to HaloTag‐CYP2D6 proteins. Three representative clones classified as higher, intermediate, and lower according to the fluorescence levels are shown in Figure 2C. RNA expression levels varied in transgenic HepaRG cell clones and were lower than in adult human liver but much higher than in WT HepaRG cells (Figure 2D). Clonal differences in red fluorescent intensity were consistent with differences in *CYP2D6* mRNA expression levels, with one exception (Figure 2E).

## 4 DISCUSSIONS

HepaRG is the only cell line that can provide an unlimited number of HLCs expressing high levels of *CYP3A4*. This is our second report of the creation of transgenic HepaRG cells. Previously, we generated transgenic HepaRG cells that can monitor *CYP3A7*‐expressing hepatoblast‐like cells and *CYP3A4*‐expressing HLCs with red and green fluorescence intensities, respectively.^11^, ^12^ In those HLCs, transcriptional induction of CYP3A4 can be detected as an increase in green fluorescence levels. In this study, we generated many HepaRG clones expressing various levels of *CYP2D6*. We found an approximate correlation between RNA expression and GFP fluorescence intensity in the CYP2D6‐iGFP expressing HepaRG clones.

HaloTag‐CYP2D6 expressing HepaRG cells can also be used to enrich the CYP2D6 enzymes and their interacting proteins during HLC differentiation. Thus, the transgenic HepaRG cells established in this study can produce abundant HLCs co‐expressing *CYP2D6* and *CYP3A4* for various experimental purposes.

In the future, we plan to measure the metabolic activity of CYP2D6 using the CYP2D6‐specific substrates of each clone to characterize transgenic HepaRG cell clones expressing various levels of the full‐length and splicing variants of CYP2D6. The results of this enzyme‐based analysis will yield basic information on whether each clone or mixture of transgenic HepaRG clones can act as human hepatocyte model cells that reflect individual differences in CYP2D6 expression. The widespread use of these cells will help solve many current issues in drug development and toxicity testing.

## DISCLOSURE

The authors declare that there is no conflict of interest, no ethical issue, and no use of clinical materials and materials from other sources.

## AUTHOR CONTRIBUTIONS

MT: research design, corresponding author, final proofreading before submission, collected data, and contributing to data analysis. SO: conducted experiments, performed data analysis and wrote the first version of the manuscript. AM, TN, YO, and MS: conducted experiments and performed data analysis.

## ETHICS STATEMENT

The authors declare that this study was performed in accordance with the research policy of Toho University.

### OPEN RESEARCH BADGES

This article has earned Open Data, Open Materials and Preregistered Research Design badges. Data, materials and the preregistered design and analysis plan are available in the article.

## Supporting information

Table S1

Method S1

## Data Availability

All data discussed in this publication are included in this manuscript. Further information and requests for data and reagents should be requested to the corresponding author, Masako Tada.
