# Supplementary material for: Transgenic HepaRG cells expressing CYP2D6 as an improved model of primary human hepatocytes
Source: Pharmacol Res Perspect. 2022 Feb 17;10(2):e00939. doi: 10.1002/prp2.939 (PMC8851295; doi:10.1002/prp2.939)
Supplement: Supplementary file 1 — Table S1 [file PRP2-10-e00939-s001.docx]

**SI Table 1.** List of PCR primers used in this study.

| Gene | Product | PCR Primers | Sequence (5' to 3') |
| --- | --- | --- | --- |
| *CYP2D6* | 65 bp | CYP2D6 ORF-F | AGGAGGAGTCGGGCTTTCT |
|  |  | CYP2D6 ORF-R | CGCTGGGATATGCAGGAG |
| *CYP2D6L* | 1,516 bp | NheⅠkozCYP2D6-F | CGGCTAGCGCCACCATGGGGCTAGAAGCACTGGTG |
| *CYP2D6S* | 1,363 bp | BamHⅠstop  CYP2D6-R | CGGGATCCCTAGCGGGGCACAGCACAAAGC |
| *CYP2D6* | 95 bp | q hCYP2D6-F | GAAGGAGGAGTCGGGCTTT |
|  |  | q hCYP3A6-R | TTTGGAAGCGTAGGACCTTG |
| *ACTB* | 67 bp | ACTB-F | ATTGGCAATGAGCGGTTC |
|  |  | ACTB-R | GGATGCCACAGGACTCCAT |
