# Supplementary material for: Transgenic HepaRG cells expressing CYP2D6 as an improved model of primary human hepatocytes
Source: Pharmacol Res Perspect. 2022 Feb 17;10(2):e00939. doi: 10.1002/prp2.939 (PMC8851295; doi:10.1002/prp2.939)
Supplement: Supplementary file 2 — Method S1 [file PRP2-10-e00939-s002.docx]

**Supporting Information**

**SI METHODS**

**1**| **Cell culture**

The medium used for HepaRG cell culture was purchased from Biopredic International, Rennes, France. The wild-type (WT) HepaRG cells at passage 19 were used. Cells were seeded at 1.5 to 2 times the recommended cell number to maintain differentiation potential. After 2 weeks of cell culture in medium 710 (Biopredic International) at 37°C and 5% CO_2_, the DMSO concentration in the medium was gradually increased from 0.1% to 0.4% 3 to 4 days before subculture. At the time of subculture, cells were gently dissociated with 0.05% Trypsin-EDTA (1×) (Thermo Fisher Scientific), and cell aggregates were seeded at 1.5 to 2 times the recommended cell number. For microscopic fluorescence imaging of transgenic cells, cells were placed in the 96-well Optical CVG plate (Thermo Fisher Scientific) at a density of 7.2×10^4^ cells/cm^2^ and cultured for several days (n=3). To produce hepatocyte-like cells (HLCs), HepaRG cells were placed in 25 cm^2^ cell culture flasks at a density of 0.5×10^6^ cells/cm^2^ and then cultured for 2 weeks in medium 710, followed by 3 days in 0.4% DMSO medium, 2 days in 1% DMSO medium, and finally 9 days in 1.7% DMSO medium. Thus, the HLC differentiation was completed in 4 weeks.

**2**| **Detailed information about the open reading frames (ORF) of *CYP2D6* isolated by PCR**

The ORF region of *CYP2D6* was amplified by PCR from hepatocyte cDNA using the primer set of CYP2D6-F and CYP2D6-R listed in the Supporting Information (SI) Table 1. The ORFs were ligated into the pGEM-T Easy vector (Promega) and sequenced. Two types of ORFs for *CYP2D6* were identified: *CYP2D6L*, 1,494 bp of DNA, which can encode the long full-length form of CYP2D6 (transcript variant 1, NM_000106.5), and *CYP2D6S*, 1,341 bp of DNA, which can encode a shorter form of CYP2D6 made by skipping exon 3 (transcript variant 2, NM_001025161.2). Since liver RNA samples from three individuals were used in this study, six different *CYP2D6* ORF sequences derived from the three sets of parental alleles should be detected. Therefore, after TA cloning and DNA sequencing of the ORF regions, one clone corresponding to *CYP2D6L* and *CYP2D6S* was selected and used for further subcloning, respectively. The nucleotide sequence of the isolated *CYP2D6* ORF was conveniently compared with the RNA sequence of *CYP2D6L*, published as NM_000106.5. As a result, the ORF regions of *CYP2D6L* and *CYP2D6S* showed a significant mutation in the 11^th^ amino acid from valine to methionine (V11M) due to the change from guanine to adenine in the 31^st^ amino acid (31G>A) of NM_000106.5 (Table 1). The allele with this amino acid substitution is called CYP2D6*35. The enzyme activity of this genotype of CYP2D6 is considered to be in a normal range^1^.

**3**| **Vector construction**

The ORF regions of the two selected TA plasmid vectors were amplified using the different ORF primer set with linkers, namely NheI kozCYP2D6-F and BamHⅠstop CYP2D6-R (Supporting Information SI Table 1). The PCR product [NheI-kozac-CYP2D6 ORF with stop codon-BamHI] was again subcloned into the TA vector. The SacII–BamHI DNA fragment was then used to generate the pCMV-*CYP2D6-IRES-GFP* expression vector shown in Figure 1. The SacII site used here is present in the TA vector. The expression vector was transformed into *E. coli* High-Efficiency DH5α Competent Cells (Takara Bio) and selectively amplified in LB medium containing ampicillin. Transgenic cells carrying this vector were easily identified by green fluorescence.

**4**| **Fluorescence image analysis**

Fluorescence microscopic images were captured using a BZ-9000 fluorescence microscope (Keyence) and an A1 confocal microscope (Nikon). Image J version 2.0.0-rc-69/1.52p software (an open-source program for image analysis provided by the National Institutes of Health) was used to calculate the mean and standard deviation of fluorescence in the total area from the images.

**5**| **Genomic PCR and RT-qPCR analysis**

Genomic PCR was performed using 20 μl of TaKaRa Ex Taq mixture (TaKaRa Bio, Shiga, Japan) and GeneAmp PCR system 9700 (Applied Biosystems). RT-qPCR was first performed by extracting total RNA from cells using the RNeasy Mini Kit (Qiagen), and cDNA was prepared using ReverTra Ace® qPCR RT Master Mix with gDNA Remover (TOYOBO). The cDNA was then amplified in 25 μl using KOD SYBR® qPCR Master Mix (TOYOBO, Osaka, Japan) and 7500 Real-Time PCR System (Applied Biosystems, Waltham, MA, USA). The relative mRNA expression levels of *CYP2D6* in transgenic HepaRG cells were determined by RT-qPCR using the primer set of q hCYP2D6-F and q hCYP2D6-R, compared to those in wild-type (WT) HepaRG cells. The *ACTB* transcripts amplified by quantitative RT-qPCR with the primer set of ACTB-F and ACTB-R was used as an internal control. The primers used in this study are listed in SI Table 1.

**REFERENCES**

1. Hicks JK, Swen JJ, Thorn CF, et al. Clinical Pharmacogenetics Implementation Consortium guideline for CYP2D6 and CYP2C19 genotypes and dosing of tricyclic antidepressants. *Clin Pharmacol Ther*. 2013;93(5):402-8.
